# Suppression of AKT Anti-Apoptotic Signaling by a Novel Drug Candidate Results in Growth Arrest and Apoptosis of Hepatocellular Carcinoma Cells

**DOI:** 10.1371/journal.pone.0054595

**Published:** 2013-01-23

**Authors:** Andrea Cuconati, Courtney Mills, Cally Goddard, Xianchao Zhang, Wenquan Yu, Haitao Guo, Xiaodong Xu, Timothy M. Block

**Affiliations:** 1 Institute for Hepatitis and Virus Research, Hepatitis B Foundation, Doylestown, Pennsylvania, United States of America; 2 Drexel Institute for Biotechnology and Virology Research, Drexel University College of Medicine, Doylestown, Pennsylvania, United States of America; 3 Enantigen Therapeutics, Incorporated, Doylestown, Pennsylvania, United States of America; 4 School of Pharmaceutical Science and Technology, Tianjin University, Tianjin, People’s Republic of China; National University of Singapore, Singapore

## Abstract

Hepatocellular carcinoma (HCC) is the third most common cause of cancer fatalities worldwide, with limited treatment options and five year survival rates of between <5 and 15%. To address this medical need, we conducted a screen of a drug-like small molecule library for HCC-selective cytotoxins. We report here the identification of a disubstituted aminothiazole termed HBF-0079, with remarkable selective toxicity for HCC-derived cell lines versus non-HCC liver lines and most other cancer lines. HBF-0079 caused irreversible growth arrest and apoptosis of the HCC lines Huh7, Hep3B, HepaRG as well as the hepatoblastoma line HepG2, with CC_50_ values from ∼0.7−7.7 µM, while more than 45 µM was needed to achieve CC_50_ values for the immortalized normal hepatocyte lines THLE-2 and PH5CH. Of the sixty cancer lines from the National Cancer Institute panel, only five exhibited >50% growth inhibition by HBF-0079. In Huh7 cells, HBF-0079 induced cell cycle arrest in G1 and concomitant apoptosis, and its effects were irreversible after removal of the compound. These observations corroborate a loss of AKT phosphorylation at the mTORC2-targeted residue S473, with concurrent loss of phosphorylation of the mTORC1 targets SK6 and 4EBP1 in Huh7 but not PH5CH cells. Finally, growth of Hep3B-derived tumors in a murine xenograft model was significantly repressed by the compound through either systemic or intratumoral administration of formulated HBF-0079. The potential for development of this drug candidate is discussed.

## Introduction

In the US, primary liver cancer is currently the fifth most common cause of cancer deaths among men, and ninth among women, with the numbers increasing yearly. In 2008 there were an estimated 21,370 new cases of liver and bile duct cancer (of which the majority are HCCs), with 18,410 deaths [1]. Worldwide, it is the third most common cancer, with approximately 695,000 fatalities reported in 2008 [2]; based on current trends and baseline models, the incidence is expected to rise to 756,000 in 2015, and 955,000 in 2030 [3]. Incidence in the US has been rising over the last 20 years, largely due to hepatitis C and nonalcoholic steatohepatitis [4], [5].

HCC is largely asymptomatic until it is very advanced, and by then treatment options are very limited. Mean survival time post-diagnosis is only 3–6 months due to the initial diagnosis usually occurring at an advanced stage [6], while mean five year survival is 5% if left untreated [7]. The gold standard treatment is surgical resection, but only 10–15% of patients can benefit because it is not indicated when the tumor has exceeded 5 cm, or has spread to multiple hepatic lobes. Liver transplantation is used with success, but is contraindicated in cases where the primary tumor has metastasized. Other treatments, such as ablation by ethanol injection into the solid tumor mass, focused ultrasound, and radiation, are practiced but rarely result in complete remission [8]. Standard cancer drugs such as doxorubicin, cisplatin, and 5- fluorouracil have been used with anecdotal success, but are limited in effectiveness [8].

In 2007, a Raf kinase inhibitor, sorafenib, became the first drug to receive FDA approval for HCC, after being demonstrated to increase post-diagnosis mean survival of patients with advanced HCC and cirrhosis from approximately 8 to 11 months [9]. Despite its limited benefit, sorafenib currently is the most effective chemotherapeutic for advanced HCC.

In light of these clinical realities, we are developing experimental treatments that selectively target HCC tissues. In the current work, we present a new chemical entity that abrogates activation of Akt and mTORC1 signaling, and results in loss of cell growth and the eventual onset of apoptosis. These effects manifest disproportionately in HCC-derived cell lines, making for the exciting prospect of a drug candidate for treatment of HCC. Although the precise target is currently being elucidated, this selectivity strongly implies that its mechanism differs from that of current HCC therapies, suggesting the possibility of a novel drug that may be used in combination with existing chemotherapeutics. We surmise that this small, chemically-tractable molecule comprises a good platform for optimization and may represent a starting point for the development of an innovative family of HCC therapeutics.

## Materials and Methods

### Ethics Statement

All animal work was conducted according to relevant national and international guidelines, including the requirements of the Association for the Assessment and Accreditation of Laboratory Animal Care International as described in the *Guide for the Care and Use of Laboratory Animals, Eighth Edition.* All animal protocols were approved by the internal Institutional Animal Care and Use Committee at Washington Biotechnology, Inc. (Baltimore MD).

### Cell Cultures and Conditions

Huh-7 [10], HeLa [11], HepG2, and Hep3B [12] and were donated by Dr. Xuanyong Lu, (Drexel University College of Medicine, Doylestown, PA). OVCAR-3 [13], MCF7 [14], and LNCAP [15] were donated by Dr. Ramila Philip (Immunotope Incorporated, Doylestown, PA). HepaRG [16] were purchased from Biopredic International (Rennes, France). MHCC97H [17] were donated by Dr. Kunwar Shailubai (Synergy Pharmaceuticals, New York, NY). THLE-2 [18] were purchased from American Type Culture Collection (Manassas, VA). PH5CH [19] were donated by Dr. Masayuki Noguchi (University of Tsukuba, Ibaraki, Japan). All cell lines were cultured and maintained in 5% CO_2_ at 37°C. Huh-7, Hep3B, HeLa, LNCAP, MCF7, and MHCC97H cells lines were maintained in DMEM/F12 with 10% Fetal bovine serum (FBS), 100 µg/mL penicillin, 100 units/mL streptomycin, and 50 µg/mL normocin. HepG2 were maintained in RPMI with 10% FBS, 100 µg/mL, penicillin, and 100 units/mL streptomycin. THLE-2 and PH5CH were maintained in BEGM with 10% FBS, 100 µg/mL penicillin, 100 units/mL streptomycin, with the following additives form the prepackaged kit: BPE, insulin, hydrocortisone, retinoic acid, transferrin, triiodothyronine, supplemented with 5 ng/ml human epidermal growth factor and 70 ng/ml phosphoethanolamine (Lonza Walkersville Inc., Walkersville, MD).

### Compound Library and Screening Assay Viability Assays

The ∼85,000-compound IHVR library includes selections from the collections of ChemDiv Inc. (San Diego, CA), Asinex Inc. (Moscow, Russia), Chembridge Inc. (San Diego, CA) and Maybridge Inc. (Cornwall, UK). The collection was assembled through “cherry picking” by computational means for diversity, solubility and drug-like qualities according to Lipinski’s Rule of Five [20], also eliminating species known to exhibit non-specific biological effects, exhibiting an average molecular weight of 345.4 Daltons, a mean cLogP of 3.42, and a mean tPSA (molecular polar surface area) of 73.56, and representing thousands of diverse pharmacophores. 64,061 of the compounds are computationally judged to be “leadlike”. The majority of the library (∼64,000) is highly diverse, with the remainder combinatorial. In addition, it includes approximately 4800 annotated compounds that are FDA- approved, have reached late stage clinical trials, or are in early stage development (LOPAC [21], [22], Microsource Discovery [23], and Johns Hopkins collections [24]). Sorafenib, doxorubicin, cisplatin and 5-fluorouracil was purchased from Selleck Chemicals (Boston, MA). All compounds are solubilized in dimethylsulfoxide (DMSO) and stored at −20°C.

In the primary library screen, Huh7 cells were plated on 96-well plates at 2.0×10^4^ cells per well to permit growth in the presence of test compounds. Compounds were prediluted and transferred to cell plates by automated liquid handling. Cells were incubated with test compounds for 72 hrs, after which culture growth and viability were assessed by addition of 50 µg/mL 3-(4,5-Dimethyl-2-thiazolyl)-2,5-diphenyl-2H-tetrazolium bromide (MTT) incubation for 4 hrs at 37°C. Solubilization buffer (0.01 M HCl, 10%SDS) was added followed by incubation at 37°C overnight. Absorbance was measured at 570 nm (reference 630 nm). The primary screening compound concentration was 10 µM at 0.5% DMSO, and 50% loss of MTT signal against DMSO-only control was scored as a hit. Secondary screening was by a four point dilution of 0.16 to 5.0 µM. Tertiary screening was by eight-point dilution in half-log steps of 0.016 to 50.0 µM, in both Huh7 and THLE-2. The concentration that is cytoxic to 50% of the cells (CC_50_) was determined using curve-fitting analysis with XLfit (IDBS, Surrey, UK).

### Colony Formation Assays

Each cell line was plated in duplicate at between 500 and 1000 cells per 60 mm plate in appropriate media. Media was replaced the next day with fresh media containing either HBF-0079 at 10.0 µM, or 0.5% DMSO. Cultures were incubated with media and compound changes every 3 days for 7–21 days, differing with rate of colony formation of each cell line. When colonies were well-formed in the DMSO control, all colonies were stained with crystal violet (10% w/v with 50% ethanol) for 10 min, rinsed and dried.

### Cell Cycle Analysis and Apoptosis Assays

Percentage of live cells was determined by harvesting at indicated time points, with 10% trypan blue (0.4% w/v in PBS) and visual counting with a hemocytometer; blue cells were deemed non-viable.

To assess cell cycle progression, Huh7 cells were treated with either HBF-0079 at 10.0 µM, or DMSO at 0.5% for 3–6 days. Cells were harvested by trypsin digestion, centrifuged at 800×G for 5 minutes, and cells were resuspended in 100 µL of 1× phosphate buffered saline (PBS), pH7.4. Cells were then added drop-wise to 50 mL of cold 70% ethanol, placed at 4°C overnight, centrifuged at 800×G for 5 min at 4°C, and washed twice in PBS. Cells were resuspended in 200 µL of Cell Cycle Guava reagent (Millipore, Billerica, MA), incubated for 30 min, and analyzed by EasyCyte Guava (Millipore) with CytoSoft 5.3.

Apoptosis was determined by Annexin-V with propidium iodide (PI) live cell staining. Huh7 cells were treated with either HBF-0079 at 10.0 µM, or DMSO at 0.5% for 3–6 days. At either time point, control cultures were incubated for 24 hr with Brefeldin A (10.0 µg/ml). Cells were harvested by limited trypsin digestion, washed once with PBS, resuspended in 100 ml of Annexin V-FITC and binding buffer per manufacturer’s instructions (BioSource International, Camarillo, CA), and stored in the dark at room temperature for 25 minutes. 0.5 µg PI was added 3 minutes before end of incubation. Cells were washed in PBS, resuspended in 400 µL binding buffer, and analyzed by EasyCyte Guava (Millipore) with CytoSoft 5.3.

### Antibodies and Immunoblotting

Monoclonal antibodies to Akt(phospho-S473), Akt(phospho-T308), total Akt, 4E-BPI (P-T37/T46), p85 S6K (P-S394), p70 S6K (P-S371), PKCα (P-S657), total PKCα, and β-Catenin were from Cell Signaling Technology (Beverly MA), and were all used at 1∶500 dilution. Monoclonal antibody to β-actin was from Millipore, used at 1∶3000 dilution. Huh7 or PH5CH cells were treated with either HBF-0079 (10.0- 0.317 µM, at 0.5% DMSO) or DMSO alone for three days in standard culture conditions. Adherent and floating cells were harvested by scraping and centrifugation, after which cell numbers were adjusted to be even, and lysed with 4× Laemmli buffer. Samples were subjected to SDS-PAGE and immunoblotting with above antibodies, and visualized by chemiluminescence (GE Healthcare, Princeton NJ ).

### In Vivo Studies

Studies on athymic nude mice (Nu/Nu, 5–6 weeks old, female) were performed at Washington Biotechnology, Inc. All animals were monitored at a minimum of once per day for pain or distress, and were anesthetized with either ketoprofen (3 mg/kg), bruprenorphine (0.1 mg/kg), or pre-surgically with isoflurane inhalation (3%). At end of studies, animals were sacrificed by sodium pentobarbital overdose (200–250 mg/kg) followed by cervical dislocation.

Pilot studies included dose escalation to determine maximum tolerated dose of HBF-0079, a single intravenous dose pharmacokinetic study, and tumor implantation trials of Huh7 and Hep3B cells, both subcutaneously and intrahepatically. The Hep3 B cell line was selected for use in a subcutaneous implantation model as this condition was found to be more consistently tumorigenic than Huh7 or orthotopic Hep3B.

Compound was formulated in normal saline (0.95% NaCl) with 22.5% wetting agent [1∶1 w/v solution of Solutol HS-15 (BASF, Florham Park, NJ)/1-Methyl-2-pyrrolidinone (NMP, Sigma-Aldrich, St. Louis MO)]. Animals were each implanted subcutaneously with 1.0×10^7^ cells in the right flank. Seventeen days later, tumors had reached an average volume of 60 mm^3^, and animals were randomly assigned to four groups, with 10 animals per group. The four arms of the principal study included: 1) Intratumor treatment with HBF-0079, 2) intraperitoneal treatment with HBF-0079, 3) intratumor treatment with vehicle, 4) intraperitoneal treatment with vehicle. Compound was administered daily for 25 days, at a dose of 8.6 mg/kg (volume of 10 ml/kg). Starting at day 7 after initial dose, mice were weighed twice weekly and tumor volume was determined by digital caliper. At day 25, mice were sacrificed and gross necropsy was performed. Tumors were harvested from a representative sample of animals from each study arm, and placed into DMEMF-12 media supplemented as described above and shipped to authors. Within 24 hours, cells were harvested from tumors by collagenase treatment, washing and subculturing for three passages each, and within 14 days HBF-0079 was tested on these lines as described above, over 8 point dilutions in triplicate, in comparison to Hep3B cells that had not been passaged in a mouse.

## Results

### Identification of HBF-0079 as a Selective Toxin for HCC Cells

A high-throughput screen of the IHVR collection was carried out, using MTT viability/growth assay [25] on the HCC-derived Huh7 cell line. Compounds that reduced MTT signal by more than 50% after three days of treatment at 10 µM were counterscreened against the immortalized human hepatocyte (HC)-derived cell line THLE-2. THLE-2 were derived through stable transfection of the SV40 large T antigen [18], and do not form tumors in athymic mice [18].

**Figure 1:**
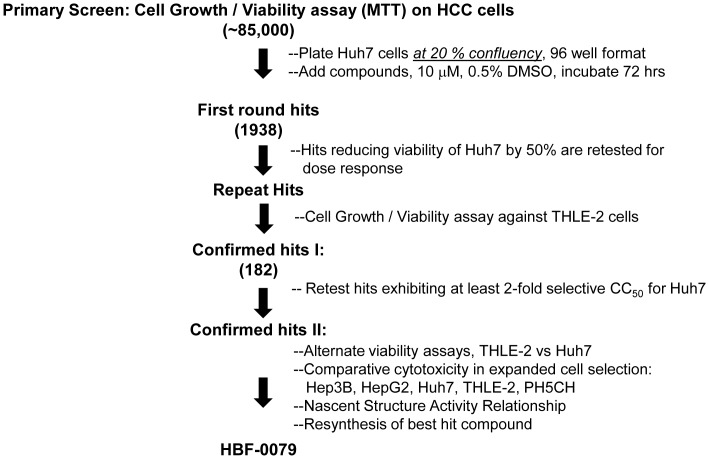
High throughput screening paradigm. Step-wise approach by which the IHVR collection compounds were tested for selective killing of HCC-derived cells. The values in parentheses represent the numbers of compounds advanced at each step.

**Figure 2:**
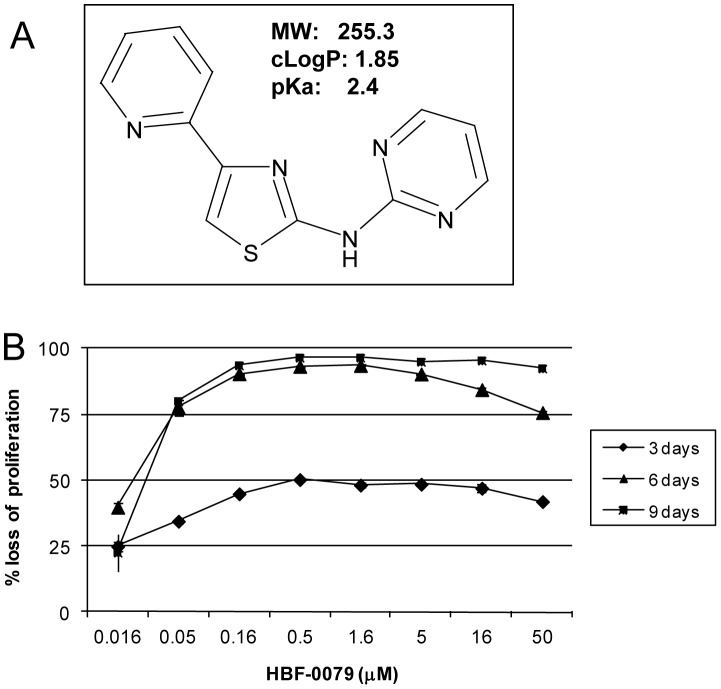
Structure and activity of HBF-0079. (A) The structure and molecular weight of compound HBF-0079, is shown with pKa and cLogP values, (ChemDraw, Cambridgesoft). (B) Percentage loss of total culture proliferation of Huh7 cells, as a function of HBF-0079 concentration vs. DMSO treatment, and duration of treatment. Log-phase Huh7 cells were cultured in the absence or presence of the indicated concentrations or 0.5% DMSO for either 3, 6 or 9 days. The percentage of viable cells remaining was then determined. Error bars indicate standard deviation of duplicate samples.

The primary screen yielded 1938 hits (Figure 1). 917 were confirmed upon re-testing, and analyzed on both Huh7 and THLE-2 at an increased range of concentrations (0.016 to 50.0 µM) to determine CC_50_. A selectivity index (SI) was calculated as the ratio of the CC_50_ on Huh7 to CC_50_ on THLE-2. 117 compounds were found to have SI values at or above 5.0, and were chosen for further investigation. After ranking hits by potency, selectivity, and evaluation for chemical tractability, a disubstituted aminothiazole resynthesis, termed HBF-0079 (Figure 2A), was resynthesized and solubilized in DMSO.

Similar to the sample selected from the library by the screen, resynthesized HBF-0079 potently inhibited proliferation of Huh7 cells, with increasing potency as length of time of compound treatment was increased. Under six days of incubation, maximal inhibition was observed, with a CC_50_ of 0.028 µM (Figure 2B). Growth curves of Huh7 cells performed under treatment with HBF-0079 or DMSO also indicated significant inhibition of proliferation, although not necessarily immediate reduction in numbers of viable cells (Figure S1).

### Cell Type Selective Toxicity of HBF-0079

To characterize the properties of this compound, inhibition of cell growth and/or viability was determined for HCC cell lines as compared to non-HCC cells.

**Figure 3:**
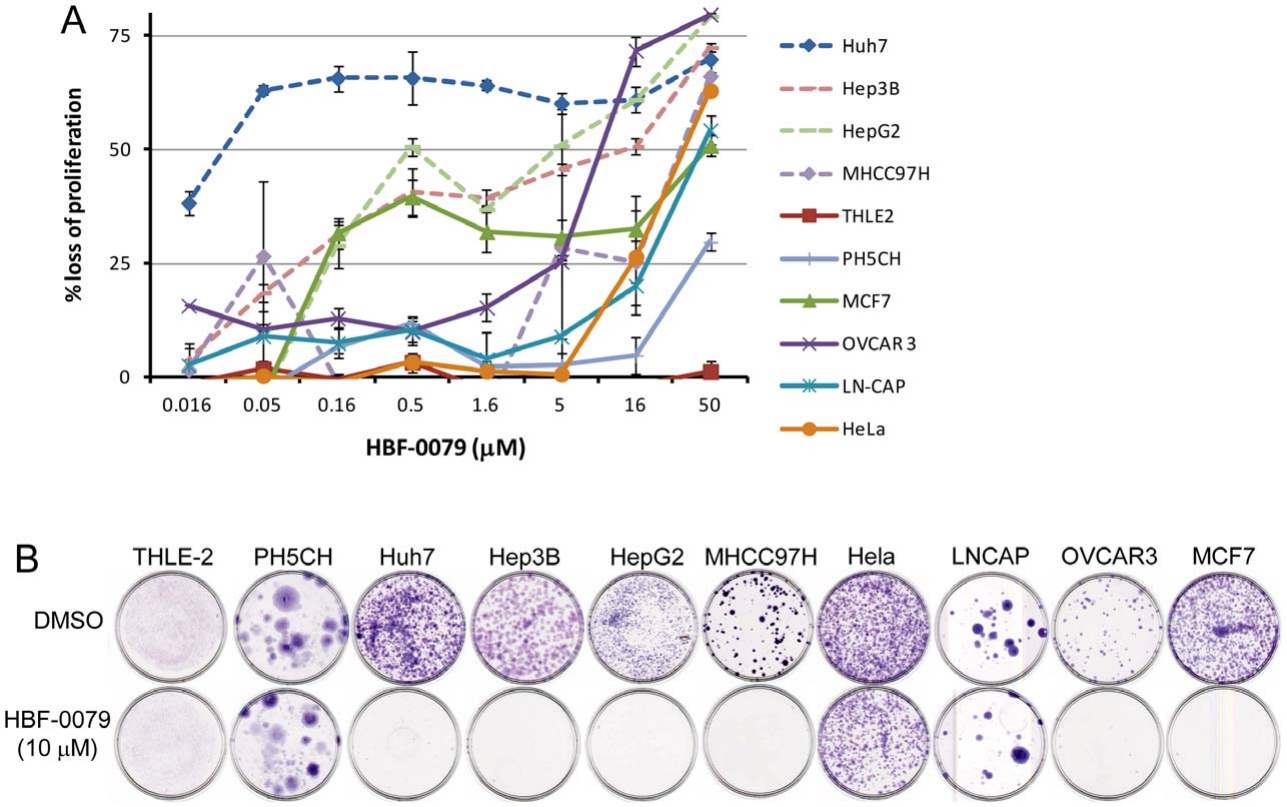
HBF-0079 cell type selectivity. The sensitivity to HBF-0079 of cell lines of different origin is compared in tissue culture. (A) Indicated cell types in log-phase were cultured in the presence of indicated concentrations of HBF-0079, or 0.5% DMSO, and percentage viability after 3 days was determined. Error bars indicate standard deviation of duplicate samples. (B) Colony formation by different cell lines (clonogenic survival) in the presence of 10 µM HBF-0079 was determined by light seeding of cells, extended culture in the absence or presence of HBF-0079 or DMSO, and staining of colonies with crystal violet.

Cytotoxicity by HBF-0079 was measured by MTT assay at varying concentrations on the HCC lines Huh7, Hep3B, HepaRG, MHCC97H; the immortalized non-malignant liver cell lines THLE-2 and PH5CH; HepG2 (hepatoblastoma); HeLa (cervical carcinoma); LNCAP (prostatic carcinoma); OVCAR3 (ovarian carcinoma); and MCF7 (breast carcinoma). HBF-0079 disproportionately inhibited three of the HCC cell lines, and interestingly also the HepG2 and MCF7 lines, while MHCC97H and OVCAR3 exhibited intermediate sensitivity. The hepatocyte lines, along with HeLa and LNCAP, were markedly resistant (Table 1 and Figure 3A). In addition, IHVR04042, an esterized derivative of HBF-0079, also exhibited selectivity for HCC lines as opposed to the immortalized hepatocytes, with 11-fold more potency than HBF-0079 (Table 1). Two–dimensional colony forming assays, which mimic clonogenic survival in a solid tumor environment, corroborated these results, with the addition of MHCC97H and OVCAR3 sensitivity to HBF-0079 in this more stringent condition (Figure 3B). The cytotoxic profile of resynthesized HBF-0079 was then compared to sorafenib, doxorubicin, cisplatin and 5-fluorouracil, with Huh7, Hep3B and THLE2 under 3-day treatment. Although HBF-0079 was less effective than other substances, it acts at lower concentrations, over a broad range (Figure S2).

**Table 1:** Cytotoxicity spectrum of HBF-0079 and the analogue IHVR-04042.

Cell Line^1^	Origin^1^	HBF-0079^2^	IHVR-04042^2^
**Huh7**	Hepatocellular carcinoma	1.1±0.84	0.097±0.068
**Hep3B**	Hepatocellular carcinoma	2.66±3.70	–
**Hepa RG**	Hepatocellular carcinoma	7.65±1.26	–
**MHCC97H**	Hepatocellular carcinoma	33.67±3.83	–
**HepG2**	Hepatoblastoma	5.71±3.6	–
**THLE-2**	SV40 LTAg-immortalized hepatocyte	44.86±12.58	>50
**PH5CH**	SV40 LTAg-immortalized hepatocyte	48.26±3.01	>50
**Hela**	Cervical carcinoma	47.23±5.32	–
**LN-CAP**	Metastatic prostatic adenocarcinoma	48.92±2.15	–
**OVCAR-3**	Ovarian adenocarcinoma	36.5±23.39	–
**MCF7**	Invasive breast ductal carcinoma	27.05±18.19	–

Cells were seeded at sub-confluence, as in text, and cultured for 3 days in the absence and presence of the indicated drug, with concentrations ranging from 0.016 to 50.0 mM. See Methods and materials for details.

1 Cell line name, and tissue of origin.

2 The concentration that is cytotoxic to 50% of the cells (CC_50_) is reported in micromolar value, with standard deviation values as determined in 4–7 experiments.

The compound was submitted to the NIH Developmental Therapeutics program for testing against the NCI-60 panel [26], and although no HCC lines are part of the panel, HBF-0079 at 10 µM for 48 hrs caused only 20% mean growth inhibition in most of the 60 non-HCC lines, as predicted by our results. The sensitivity of MCF7 was confirmed in that testing with 89% growth inhibition, along with that of T47D breast carcinoma (66%) and NCI/ADR-RES ovarian carcinoma lines (66%) (Results not shown). These results corroborate our own observations, and suggest that HBF-0079 functions through a cell-type specific mechanism.

### HBF-0079 Induces non-reversible Growth Arrest and Subsequent Death in HCC Cells

To determine whether the selective cytotoxicity of this compound could be reversed by removal, Huh7 cells were treated with HBF-0079 at 10 µM for six days with media changes and fresh compound or DMSO alone added every two days. After six days, compound was either 1) removed, and fresh media containing DMSO was added, 2) treatment was continued with fresh compound, or 3) mock treatment (DMSO) was continued, each condition extended for 6 more days, with refreshment. Percentage of dead versus total cells was determined every day.

When compound was withdrawn (cmpd/DMSO), total cell number did not recover for the entire six days after removal, paralleling that of the sample under continued treatment (cmpd/cmpd) (Figure S3A). Correspondingly, the percentage of dead cells was high (50–80%) under both withdrawal and continued treatment, indicating that treatment was not reversible (Figure S3B). The mock treated sample (DMSO/DMSO) exhibited high total cells and a low percentage of dead cells. Interestingly, when the compound treatment was initiated on a confluent culture after an initial 6 day mock incubation (DMSO/cmpd), the total cell number did not reduce appreciably, and the percentage of dead cells also did not increase. Similarly, when compound treatment was carried out without preincubation (no treatment/cmpd), no loss in total cell numbers was observed, but there was an increase in the percentage of dead cells after 3 days (Figure S3A and B). These data are consistent with an initially cytostatic effect, with subsequent cell death.

**Figure 4:**
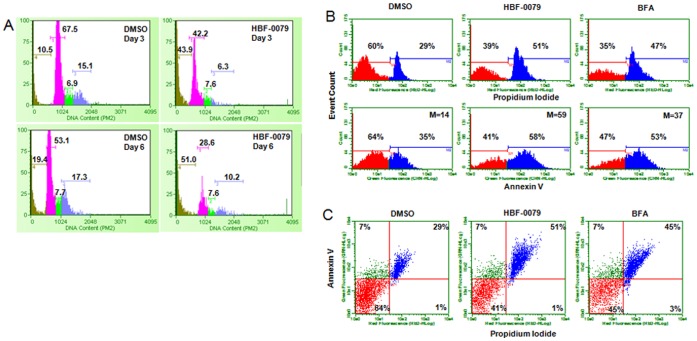
HBF-0079 induces cell cycle arrest and apoptosis in HCC cells. (A) Log-phase Huh7 cells were incubated in the absence or presence of 10 µM HBF-0079 or 0.5% DMSO for up to 6 days. Cell cycle phase distribution was determined at days 3 and 6 by PI labeling of total DNA content flow cytometry, and analysis through the Guava Cell Cycle Assay software module. From left to right, colored areas indicate SubG_0_,/G_1_, G_0_,/G_1_, S, G_2_/M phases. Cell population percentages in each phase are indicated above peaks. (B) PI vs annexin V staining of Huh7 cells treated with DMSO, HBF-0079, or BFA for 6 days. Top panels depict histograms of staining intensity vs cell/event count; bracketed areas indicate percentage of cells positive for strong vs weak staining with PI or Annexin V as indicated by the X axis. Shift in toward stronger Annexin V staining is indicated by median value (M) calculated from a linear axis (not shown). (C) Dot plot analysis of PI vs Annexin V co-staining of data set from (B), with percentage of cells in each quadrant indicated. Cells in upper right quadrant are positive for both PI and Annexin V, indicating onset of apoptosis.

To determine the effects of HBF-0079 on cell cycle progression, Huh7 cells were treated with at 10 µM for up to six days with refreshment every two days, and analyzed for cell cycle phase distribution at days 3 and 6 by propidium iodide (PI) staining and flow cytometry. In comparison to DMSO treatment, HBF-0079 exposure resulted in a shift of the cell population from G_1_ to sub-G_1_/G_0_ with a concomitant reduction of the cell population in G_2_, seen at day 3 and progressing further at day 6 (Figure 4A). This pattern is characteristic of cell cycle arrest in G_1_ followed by cell death.

We investigated the implications of these observations by determining whether HBF-0079 can induce apoptosis in HCC cells. Huh7 cells treated with 10.0 µM for 1, 6, and 9 days were analyzed by annexin V staining, with cell viability monitored by PI staining. Brefeldin A (BFA, 1.0 µM) was added to positive control samples 24 hrs prior to each collection. HBF-0079 treatment resulted in PI-positive corresponding with annexin V staining, indicating death by apoptosis (Figure 4B and C). HBF-0079 treatment resulted in significant percentage of apoptotic cells from three to six days of incubation, while mock (DMSO) treatment surprisingly resulted in measurable, albeit lower, percentage of dead cells, likely due to overgrowth and subsequent detachment of senescent cells. Analysis at one and nine treatment days revealed a trend in the early onset of apoptosis, with a decrease in Annexin V staining at between six and nine days (Figures 4B/C and S4). Since treatment of log-phase, growing cell cultures with HBF-0079 essentially “freezes” overall total cell numbers (Figures S1 and S3), it is likely that the compound results in initial block in cell growth, followed by onset of apoptosis.

Overall, these observations indicate that HBF-0079 induces a block in proliferation, followed by eventual loss of viability. This cytostatic effect is not likely to underlie the selectivity of the compound, because HeLa and other rapidly proliferating lines were insensitive. Most importantly, removal of the compound does not rescue cell growth and viability, suggesting that an efficacious dosage regimen may be relatively infrequent.

### HBF-0079 Modulates Signaling through AKT-dependent Pathways

**Figure 5:**
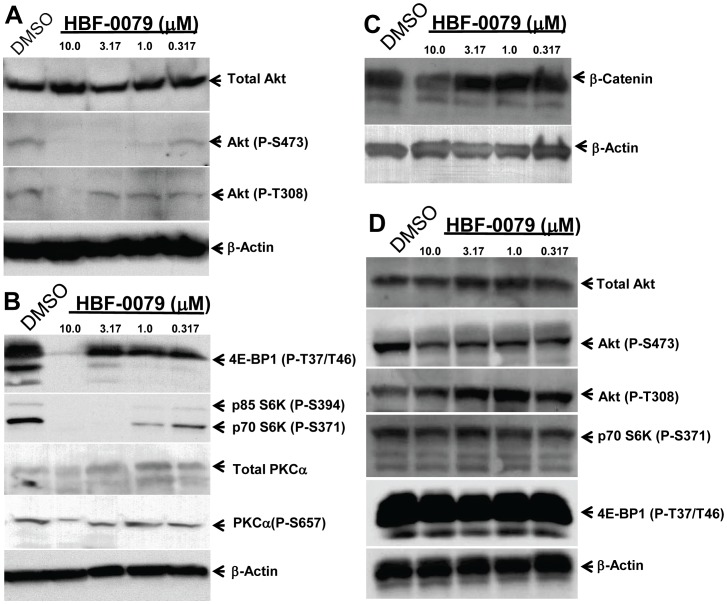
HBF-0079 modulates cell-growth and anti-apoptotic signaling through AKT and mTOR. Log-phase Huh7 were cultured for 3 days in the absence or presence of HBF-0079 at indicated concentrations, or 0.5% DMSO, lysates were analyzed by Western blotting using Abs specific for (A) total Akt, Akt phosphorylated at S473, Akt phosphorylated at T308, or β-Actin; (B) mTOR targets 4E-BP1 phosphorylated at T37/T46, S6K phosphorylated at S371/394, total PKCα, PKCα phosphorylated at S657, and β-Actin; (C) β-catenin and β-Actin; (D) Log-phase PH5CH cells were treated, cultured and analyzed for AKT, 4E-BP1 and S6K as in (A,B). Details in Methods.

Inappropriately activated mitogenic and anti-apoptotic signaling by constitutive activation of AKT kinase is one of the more common oncogenic mechanisms found in HCC [27], [28], [29], [30]. In Huh7 cells in particular, AKT is strongly activated though phosphorylation at serine 473 [31]. We investigated whether the cytostatic and cytotoxic effects of HBF-0079 were correlated with changes in AKT-dependent signaling. Incubation of Huh7 cells with varying concentrations of HBF-0079 for 3 days resulted in reduced phosphorylation at S473 at 1.0–2.5 µM, analyzing equal numbers of cells (Figure 5A). Because S473 is predominantly a target of the mTOR complex C2 [32], we examined phosphorylation states of mTOR targets. HBF-0079 treatment resulted in loss of phosphorylation of the mTORC1 targets 4E-BP1 and S6K, which would be predicted to lead to overall shutoff of cap-dependent translation, cell growth arrest and apoptosis [32]. Paradoxically, phosphorylation of the mTORC2 target PKCα was only minimally affected, unlike AKT at S473, suggesting that mTORC1 is more directly affected by HBF-0079 treatment than mTORC2 (Figure 5B). This may be due to the loss of feedback inhibition of mTORC2 through loss of S6K activation [33], , with downregulation of S6K phosphorylation by mTORC1 ultimately stimulating mTORC2 activity. Alternatively, phosphorylation at S473 may be regulated by additional factors besides mTORC2, as has been proposed [32].

Destabilization of β-catenin was only observed at the highest concentration of HBF-0079 used, indicating that it is likely to be a indirect effect of the compound, and that the Wnt pathway is not targeted (Figure 5C).

Interestingly, in the HBF-0079-insensitive PH5CH line only slight reduction of Akt S473 phosphorylation was noted, and no effect on 4E-BP1 and S6K phosphorylation was seen, correlating with the compounds lack of toxicity in these cells (Figure 5D). Although none of these factors are necessarily direct molecular targets of HBF-0079, these observations suggest the compound inhibits uncontrolled proliferation and induces apoptosis through inhibition of AKT-dependent signaling and mTOR regulation of protein synthesis. Interestingly, *in vitro* screening of a panel of over 300 kinases has indicated that HBF-0079 is not a direct inhibitor of AKT, mTOR, PDK1, or PI3K, (results not shown). Thus, it is possible that the target of HBF-0079 affects Akt/mTOR signaling in an indirect manner.

### Inhibition of Tumor Growth *in vivo* by HBF-0079

**Figure 6:**
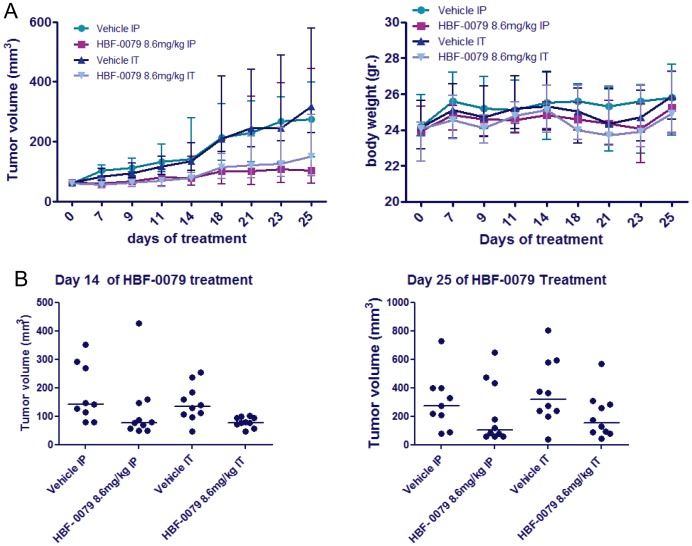
Effects of HBF-0079 in a xenograft model of HCC. Mice bearing subcutaneous Hep3B tumors were treated with either vehicle alone, or vehicle supplemented with HBF-0079, once a day for up to 25 days via IP, or IT injection. At the indicated times tumor volume (A) and animal weight were determined. Median values with interquartile error range are shown. (B**)** Scatter plots of tumor size distribution at 14 and 25 days of treatment. Median values are represented by horizontal bar.

Following formulation, the pharmacokinetic (PK) properties and maximum tolerated dose of HBF-0079 were determined in a murine model. The compound was well-tolerated after intraperitoneal (IP) administration up to 16 mg/kg, and serum half life was found to be approximately 45 minutes after reaching maximum concentration (results not shown). Subsequently, the compound was administered to athymic “nude” mice harboring subcutaneous Hep3B-derived tumors by intraperitoneal (IP) injection, or by direct intratumor (IT) injection in different groups. IT injection simulates chemoembolism treatment, while IP simulates systemic exposure. Tumor volume was determined by caliper twice weekly after an initial 7 days. As early as 9 days of treatment, and continuing for 25 days, HBF-0079 significantly repressed median tumor growth through either IT or IP injection, and the majority of animals exhibited reduced to absent tumor growth, or complete tumor regression. No apparent changes in body weight were noted in any group (Figure 6A); in contrast, sorafenib was reported to require at least 21 days for efficacy in the same model, and with significant weight decrease [36]. Individual animals exhibited varied responses in tumor size, with a small minority showing no response (Figure 6B). Necropsies at 25 days found no gross abnormalities in organs systems of any group. Cell lines derived from representative large and small tumors were isolated, expanded and tested for sensitivity to HBF-0079; these were found to still exhibit general sensitivity similar to the parental Hep3B line (results not shown), suggesting that induction of physiological clearance mechanisms, such as cytochrome P450 (CYP) family of enzymes, may have resulted in lower compound exposure. Another possibility is variations in the degree of vacularization of the tumors. These are common properties of HCC xenograft models, and were also observed with sorafenib treatment of Hep3B tumors [36]. To address these points, future studies will use intrahepatic orthotopic implantation of the HCC cells, as well as monitoring for CYP induction. Overall, despite the fact that a direct comparison between these two compounds cannot be made from two separate studies, these observations do indicate that HBF-0079 can exhibit antitumor activity that is superior to that reported with sorafenib against HCC cells in an *in vivo* system.

## Discussion

The distinct gene expression patterns of clinically indistinguishable HCC tumors suggests that the molecular mechanisms of HCC establishment and maintenance vary widely [37], [38], [39], [40], [41], underlying the difficulty in chemotherapeutic treatment of this cancer; ostensibly, different agents would act on different pathways, and thus would only be effective in a subset of HCC cases. In fact, traditional chemotherapeutics like cisplatin and doxorubicin have shown anecdotal efficacy, but have proven disappointing in larger studies. Sorafenib extends survival by an average of four months, but its efficacy is offset by significant side effects. It is clear that the available arsenal of HCC therapies would benefit from the inclusion of novel chemotherapeutic agents that would improve efficacy and tolerability. To that end, we have focused our early-stage drug discovery efforts on identification of compounds with tumor cell type-specificity; with sufficient specificity, a drug candidate may be administered without side effects at high enough levels to provide sufficient anti-tumor effect. As a result, we have identified a compound with remarkable selectivity, but with considerable efficacy in both *in vivo* and tissue culture models.

HBF-0079 induces cell cycle arrest and apoptosis with an exquisite degree of selectivity for several HCC lines versus two non-HCC hepatocyte lines, and with activity against some human cancers lines but not others, clearly demonstrating that the compound acts through a cell type-specific mechanism rather than as a general cytotoxin. *In vivo*, it exhibits anti-tumor effects with high tolerability.

The compound disrupts mitogenic and anti-apoptotic signaling through the Akt/mTOR axis, correlating with the phenotypic observations of growth arrest and apoptosis. Akt/protein kinase B inhibits apoptosis in a variety of tissue culture and animal models [42], [43], is often overexpressed in HCC [27], [28], [29], [44], [45], and is activated by sequential phosphorylation at two sites: 1) S473 by mammalian target of rapamycin complex 2 (mTORC2), autophosphorylation, and by likely other kinases; and 2) T308 by phosphoinositide-dependent kinase 1 (PDK1). Since mTOR cycles between mTORC1 and mTORC2, a gain of mTORC1 activity through Akt signaling could hypothetically favor formation of mTORC2, and further activation of Akt by phosphorylation at S473. Conversely, loss of Akt phosphorylation at S473, as induced by HBF-0079, would result in reduced mTORC1 signaling, and thus reduced cap-dependent translation [32]. Thus, inhibition of Akt signaling would result in increased apoptotic potential, a decrease in cytoskeletal assembly and cap-dependent translation, and loss of mitogenic signaling through Akt-regulated WNT/β-Catenin and NFκB target genes. *In vitro* screening suggests that HBF-0079 does not act upon these kinases directly (results not shown), suggesting that the molecular target of the compound may act upon this pathway indirectly.

Perhaps the most interesting property of HBF-0079 is its selectivity. HCC and hepatoma-derived cell lines were up to 40 times more sensitive to HBF-0079 than were non-malignant liver cell lines, and some non-HCC lines. This observation clearly indicates that the compound is not a general cytotoxin, and is likely to be functioning through cell-specific mechanisms. This selectivity could indicate one or more circumstances: 1) The molecular target(s) may be expressed differentially in certain tissue and/or tumor types; 2) HBF-0079 is metabolized to an active form, or is degraded to an inactive form, only in certain tissue and/or tumor types; 3) Assuming an intracellular target, passage of the compound past the plasma membrane is restricted in certain cell types. Of particular interest is that although Huh7, Hep3B and HepG2 differ in their profiles for p53 and β-catenin mutation and/or activation [46], they are all similarly sensitive to HBF-0079.

Several studies have attributed a variety of biological activities to disubstituted aminothiazoles, including induction of autophagy in renal carcinoma cells [47], inhibition of potassium ion channels [48], and inhibition of prion pathogenesis [49], [50]. The relationship between these observations and our own results is currently being investigated, but preliminary work indicates that the trend we are observing in the structure-activity relationship (SAR) studies diverges from that in at least one of these reports [48]. This would suggest that the effects prompted by HBF-0079 and its derivatives work through an alternate mechanism from ion channels inhibition, but it also possible that the SAR divergence may be due to binding to a different ion channel, and thus that particular mechanism remains a possibility.

Regardless of the mechanism, the selectivity of HBF-0079 that is observed in cell culture translates to high tolerability in an *in vivo* murine model. Although a complete toxicity profile with serum chemistry and blood cell counts has not yet been performed, it is likely that the lack of compound-specific adverse events, along with maintenance of body weight over a 25-day dosing period, indicates that HBF-0079 is well-tolerated. Thus, we have achieved our initial goal of finding a new compound that is non-toxic and efficacious in the reduction of HCC solid tumors. In future reports we will detail progress in the development of HBF-0079 and derivatives; ongoing structure-activity relationship studies have already identified relevant features of the molecule, and resulted in two compounds that retain selectivity and lower CC_50_ by approximately 10-fold in Huh7. In addition, putative molecular targets are being analyzed as result *of vitro* screening of a panel of over 300 kinases, and will be reported on in the future.

## Supporting Information

Figure S1 HBF-0079 arrests growth of an HCC-derived cell line. Log-phase Huh7 cells were plated at ∼10% confluency and incubated with HBF-0079 or DMSO as indicated. Growth curve was generated by trypan blue assay and total cell counting by hemocytometer.
(TIF)

Figure S2 Hepatic cell type selectivity of HBF-0079 and clinical chemotherapeutics. Percentage loss of total culture proliferation as a function of HBF-0079, sorafenib, cisplatin, doxorubicin, or 5-fluorouracil concentration vs. DMSO treatment. Log-phase Huh7, Hep3B or THLE-2 cells were cultured in the absence or presence of the indicated concentrations of HBF-0079, 0.5% DMSO, or indicated compounds for 3 days. The percentage of viable cells remaining was determined by MTT assay.
(TIF)

Figure S3 Effects of treatment with HBF-0079 on HCC cells are irreversible. Log-phase Huh7 cells were cultured in the absence or presence of 10 µM HBF-0079 or 0.5% DMSO for 6 days, after which treatment was continued, discontinued or initiated for another 6 days, as described in text. Error bars indicate standard error percentage based on multiple representative cell count samples. (A) Total number of live and dead cells at each day past initial 6-day treatment, as determined by trypan blue assay and hemocytometer counting. (B) Cell viability was determined in (A) and expressed as percentage dead cells versus total cells.
(TIF)

Figure S4 HBF-0079 induces apoptosis in HCC cells over short and long duration treatment. PI vs annexin V staining of Huh7 cells treated with DMSO, HBF-0079, or BFA for either 1 or 9 days, as in Figure 4B. Top panels depict histograms of staining intensity vs cell/event count. Bottom panels depict dot plot analysis of PI vs Annexin V co-staining.
(TIF)
