# Supplementary figures and images for: Suppression of AKT Anti-Apoptotic Signaling by a Novel Drug Candidate Results in Growth Arrest and Apoptosis of Hepatocellular Carcinoma Cells
Source: PLoS One. 2013 Jan 23;8(1):e54595. doi: 10.1371/journal.pone.0054595 (PMC3552860; doi:10.1371/journal.pone.0054595)

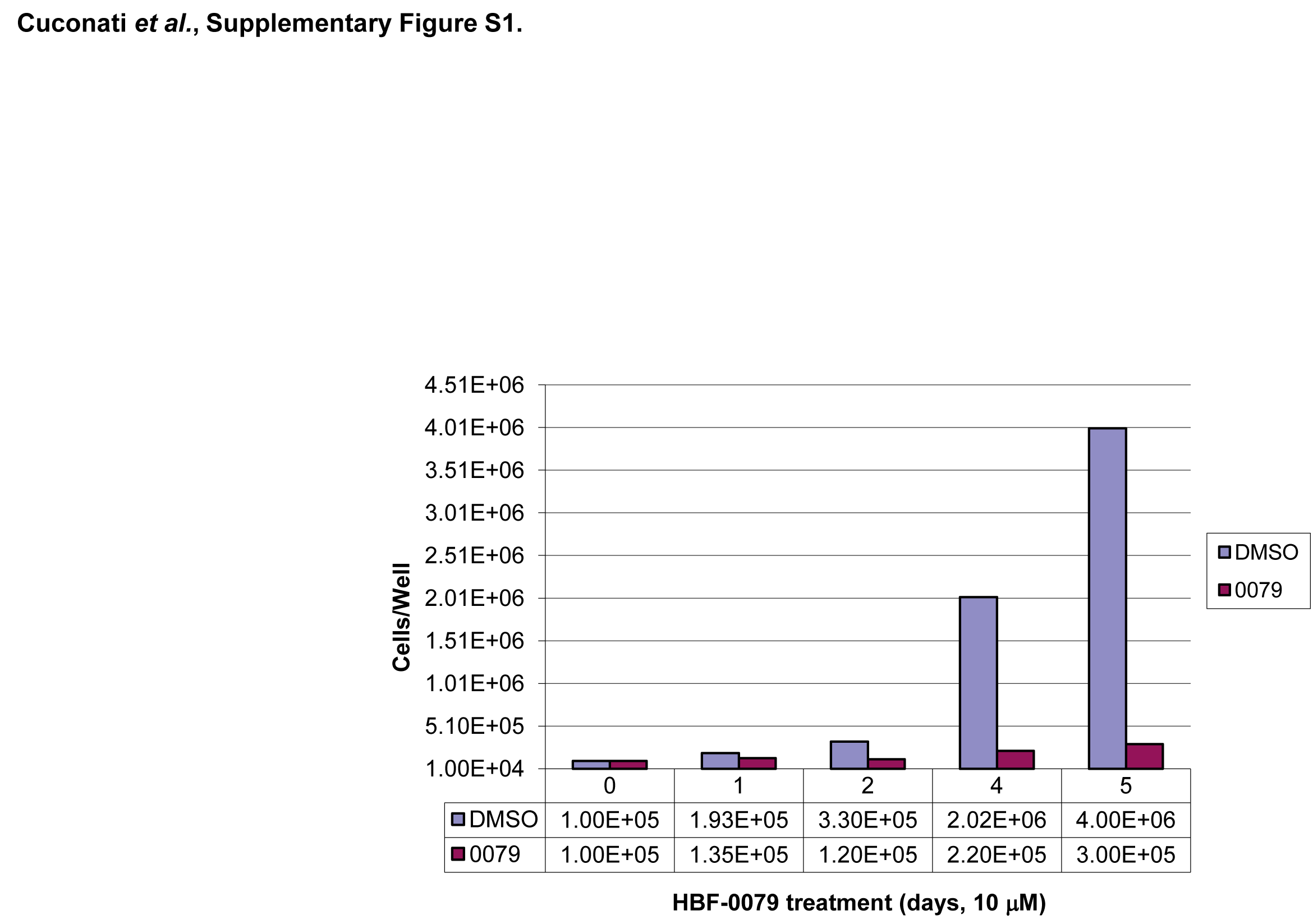

Supplement: Figure S1 — HBF-0079 arrests growth of an HCC-derived cell line. Log-phase Huh7 cells were plated at ∼10% confluency and incubated with HBF-0079 or DMSO as indicated. Growth curve was generated by trypan blue assay and total cell counting by hemocytometer. (TIF) [file pone.0054595.s001.tif]

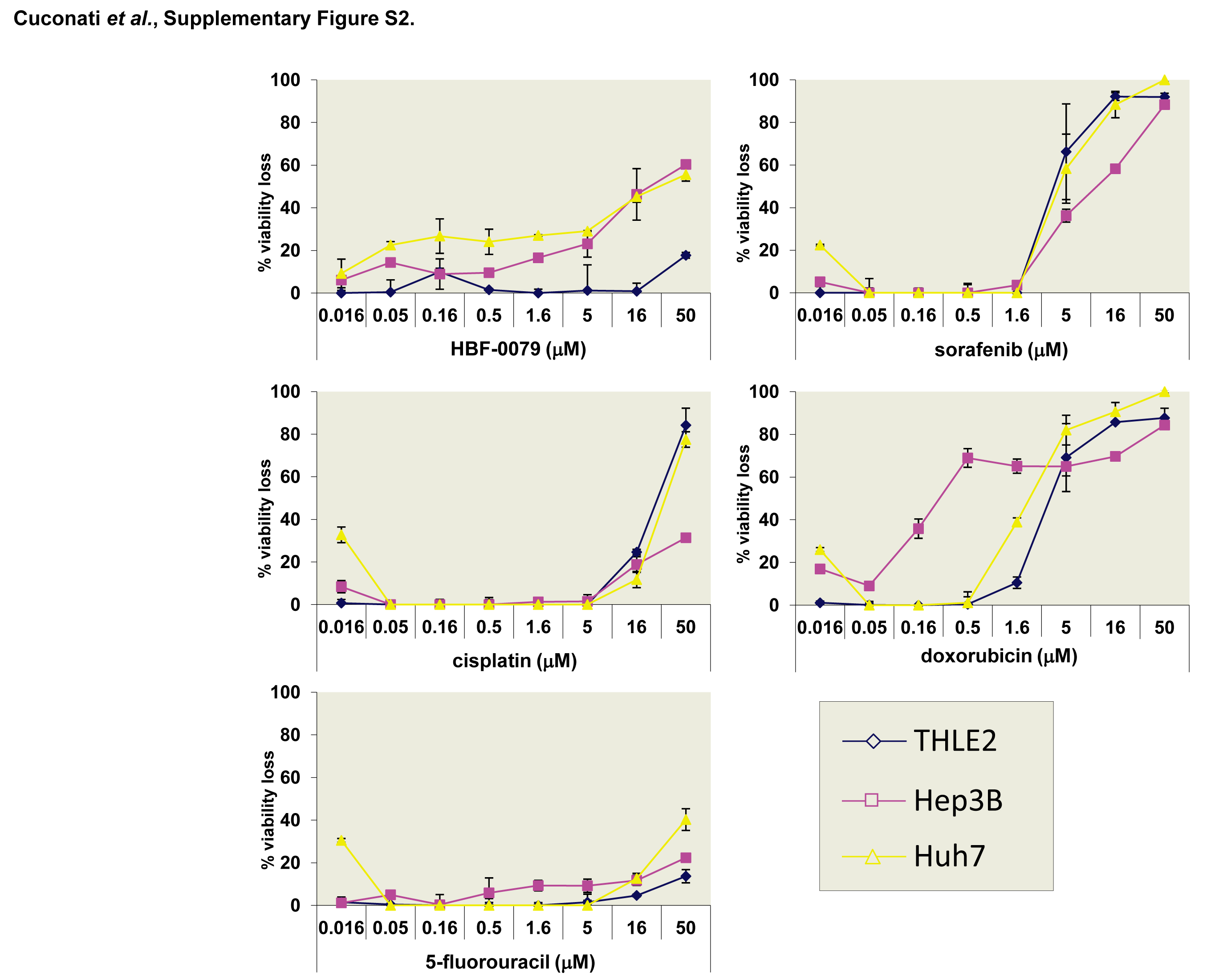

Supplement: Figure S2 — Hepatic cell type selectivity of HBF-0079 and clinical chemotherapeutics. Percentage loss of total culture proliferation as a function of HBF-0079, sorafenib, cisplatin, doxorubicin, or 5-fluorouracil concentration vs. DMSO treatment. Log-phase Huh7, Hep3B or THLE-2 cells were cultured in the absence or presence of the indicated concentrations of HBF-0079, 0.5% DMSO, or indicated compounds for 3 days. The percentage of viable cells remaining was determined by MTT assay. (TIF) [file pone.0054595.s002.tif]

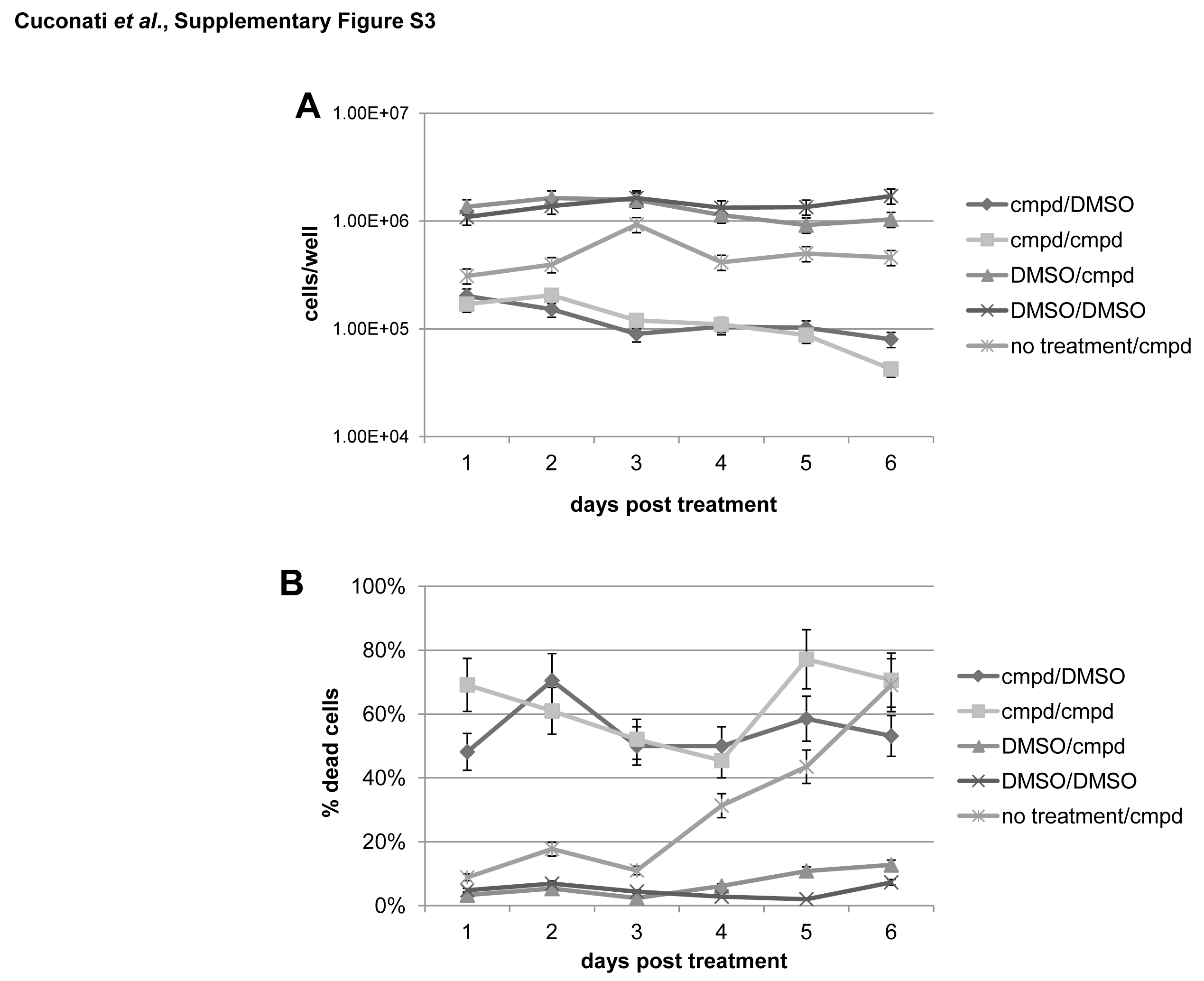

Supplement: Figure S3 — Effects of treatment with HBF-0079 on HCC cells are irreversible. Log-phase Huh7 cells were cultured in the absence or presence of 10 µM HBF-0079 or 0.5% DMSO for 6 days, after which treatment was continued, discontinued or initiated for another 6 days, as described in text. Error bars indicate standard error percentage based on multiple representative cell count samples. (A) Total number of live and dead cells at each day past initial 6-day treatment, as determined by trypan blue assay and hemocytometer counting. (B) Cell viability was determined in (A) and expressed as percentage dead cells versus total cells. (TIF) [file pone.0054595.s003.tif]

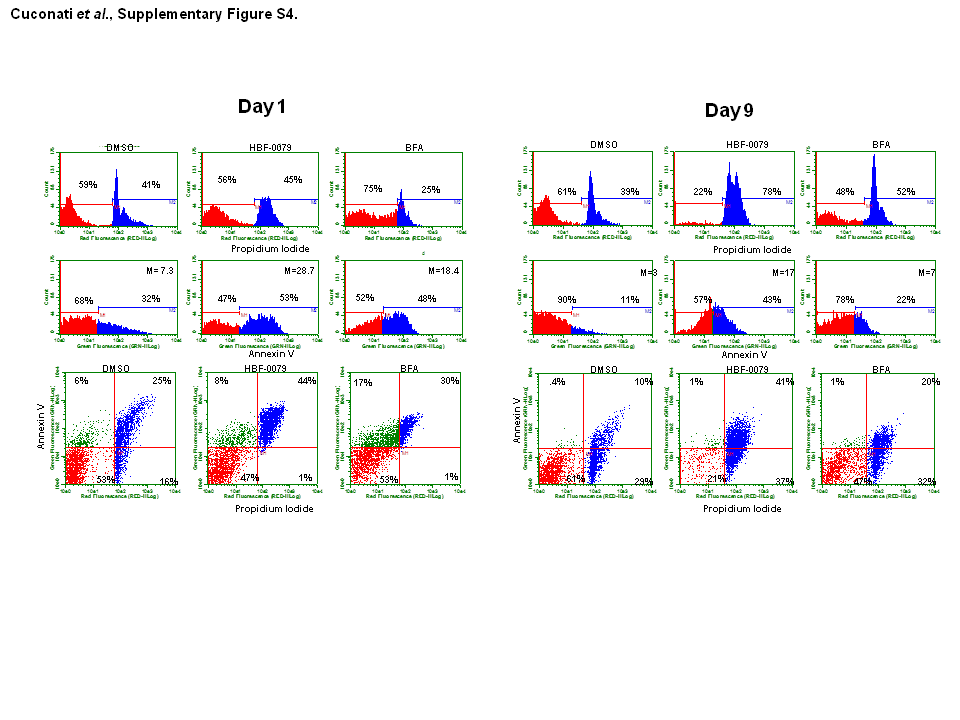

Supplement: Figure S4 — HBF-0079 induces apoptosis in HCC cells over short and long duration treatment. PI vs annexin V staining of Huh7 cells treated with DMSO, HBF-0079, or BFA for either 1 or 9 days, as in Figure 4B. Top panels depict histograms of staining intensity vs cell/event count. Bottom panels depict dot plot analysis of PI vs Annexin V co-staining. (TIF) [file pone.0054595.s004.tif]
